# A potential pathway for identifying hypertension among urban residents aged 60+ years in China: the role of health insurance

**DOI:** 10.3389/fpubh.2024.1420465

**Published:** 2024-05-15

**Authors:** Yu Dou, Hongmei Guo, Sijun Liu, Huiqing Xu, Fengli Li, Wanying Tao, Shifen Jia, Siyu Tian, Tianrui Deng, Yaqing Xiong, Fei Xu

**Affiliations:** ^1^Geriatric Hospital of Nanjing Medical University, Nanjing, China; ^2^School of Public Health, Nanjing Medical University, Nanjing, China; ^3^Nanjing Municipal Center for Disease Control & Prevention, Nanjing, China

**Keywords:** identification, health insurance, hypertension, Chinese, older resident

## Abstract

**Background:**

Identification is the first step for treatment of hypertension. However, the awareness rate of hypertension was not high globally. This study aimed to examine the potential role of health insurance for early-identifying hypertension among urban older residents in China.

**Methods:**

In this cross-sectional study, urban residents aged 60+ years were chosen from Nanjing municipality of China in 2018. The outcome measure was hypertension status (“no hypertension,” “diagnosed hypertension” or “un-diagnosed hypertension”). Independent variable was health insurance (“Urban Employee Basic Medical Insurance scheme, UEBMI” or “Urban Resident Basic Medical Insurance scheme, URBMI”). Logistic regression models were introduced to estimate odds ratio (OR) and 95% confidence interval (CI) to examine the association between health insurance and hypertension.

**Results:**

Totally, 19,742 participants completed the study. Among overall, URBMI and UEBMI participants, 47.2% (95%CI = 46.5, 47.9%), 38.4% (95%CI = 37.3, 39.6%) and 52.1% (95%CI = 51.2, 53.0%), separately, were diagnosed with hypertension, while the prevalence of un-diagnosed hypertension was 12.7% (95%CI = 12.2, 13.2%), 18.5% (95%CI = 17.6, 19.4%) and 9.6% (95%CI = 9.1, 10.1%), respectively. For overall participants, those with UEBMI were more likely to have hypertension identified (OR = 1.20; 95%CI = 1.11, 1.29) and at lower odds to experience un-diagnosed hypertension (OR = 0.68; 95%CI = 0.61, 0.76) compared to their counterparts with URBMI after control for potential confounders. Moreover, such associations of health insurance with diagnosed and un-diagnosed hypertension were also observed among participants stratified by age and gender.

**Conclusion:**

Favorable health insurance may be a pathway for identifying hypertension among urban older residents in China. This study has important public health implications that hypertension may be identified early through favorable health insurance policies for older residents in China.

## Introduction

Hypertension (HTN) is a major contributor to cardiovascular diseases (CVDs) and the leading cause of premature death worldwide (1). It has been documented by World Health Organization (WHO) in 2021 that approximately 1.28 billion adults aged 30–79 years experienced HTN globally (1), and this estimate would be about 1.56 billion by 2025 (2). On the other hand, among overall HTN individuals only 54.0% were diagnosed and further only 21.0% of these identified HTN patients had their blood pressure (BP) under control (1). For China, the diagnostic rate of HTN was just 41.0% based on a nationally representative data collected in 2018 (3), which was much lower than the global average level (1). From a public health perspective, the earlier HTN individuals are identified and the more HTN patients have their blood pressure under control, the much better for HTN treatment.

China, the most populous country in the world, has been witnessing much higher HTN prevalence among residents aged 60+ years (53.2%) than adults aged 35–59 years (26.1%) (4, 5). It implies that the disease burden caused by HTN was much heavier for older people than those adults aged 35–59 years in China. Moreover, the proportion of people aged 60+ years increased from 10.3% in 2000 to 18.7% in 2020, showing a rapid aging of population in China (6). Therefore, it is a priority for China to improve the diagnostic rate of HTN among residents, especially the older people. In other words, it is an urgent need to identify individuals with HTN as early as possible in the campaign against HTN at the population level in China.

With efforts to enhance diagnostic rate of HTN, experts called for doctors to assess BP for individuals aged 35+ years at their each visit to hospitals in early 1990s in China (7). Shanghai Municipal Health Bureau launched a regulation that BP should be measured for all people who visited grade-one hospitals (primary care/community health service centers) in Shanghai since July 1, 1999 (8). Surprisingly, 35.8% of outpatients were identified with HTN under this regulation during the starting half year (from July one to December 31 of 1999) in Shanghai (8). Furthermore, such an approach for promoting HTN identification through BP measurement at patients’ visits to hospitals was examined to have good feasibility in addition to its effectiveness in different regions of China (9). Thus, the Central Government of China in 2012 formally issued a policy that doctors shall provide BP measurement for each patient at his/her visit to general hospitals or community-level primary care centers (10). Consequently, each patient would have his/her BP assessed at each visit to a hospital/primary care center by a doctor after 2012 in China.

Reasonably, the more frequently an individual visits hospitals/primary care centers, the more likely he/she will have his/her BP assessed by a doctor. In such a case, individuals with HTN can be diagnosed as early as possible. Interestingly, health insurance has been investigated to be positively associated with visit to hospitals/primary care centers among residents, particularly urban inhabitants in China (11–19). Moreover, health insurance was also examined in a positive relation to diagnosis of HTN for age-specific adults in USA and China (20–23). Therefore, it may be practicable and effective for identifying HTN through improving health insurance coverage and level.

Currently, there are two health insurance schemes for urban residents in China. One is the Urban Employee Basic Medical Insurance scheme (UEBMI) which was launched in 1999 (24). All urban employees were required for a compulsory enrollment with UEBMI and, of course, would have UEBMI even after retirement (24). The other is the Urban Resident Basic Medical Insurance scheme (URBMI) that was established in 2007 (25). URBMI was recommended for those residents who had no formal employment contract or were unemployed, including kids and students (25). Compared to URBMI, UEBMI stipulates a higher financing level and, of course, a higher reimbursement rate, and consequently has more benefits for the insured in China (26). With continuous efforts made by the Central Government of China, almost a universal coverage of health insurance has been achieved recently (95% rural and 100% urban residents covered by either UEBMI or URBMI at the end of 2017) in China (27).

Considering that: (1) each urban resident holds a health insurance scheme (UEBMI or URBMI), (2) health insurance is positively associated with visit to hospitals, (3) an individual will have his/her BP measured by a doctor when he/she visits a hospital/primary care center, and (4) UEBMI has more benefits compared to URBMI for the insured, we hypothesized that, compared to their counterparts with URBMI, individuals with UEBMI may have higher odds of being identified early if they have HTN in China. To test this hypothesis, we conducted a study to examine the associations of health insurance with diagnosed and un-diagnosed HTN, separately, among urban residents aged 60+ years in regional China.

## Methods

### Study design and participants

This was a cross-sectional study, conducted in Nanjing municipality, a typical megacity in eastern China, in 2018. Nanjing had five urban and six suburban administrative districts and approximately 8.43 million registered inhabitants, with 20.9% aged 60+ years, in 2018 (28). To be eligible for the study, a participant must be: (1) a resident registered in one of the five urban districts of Nanjing, (2) 60+ years old, (3) without psychiatric disorders, and (4) without literal/cognitive problems.

A multi-stage sampling method was applied to select participants. The study sample size was determined to be sufficient at the district level. The number of participants within a single district was estimated with consideration of the sampling approach employed, the presently-available prevalence (22.9%) of un-diagnosed HTN among urban older residents (3), expected response rate (85%), statistical power (90%), and stratified analysis expected (3 subgroups of age). Consequently, the district-level sample size should be about 3,402, resulting in that approximately 17,010 participants would be recruited from all the five urban districts at the municipality-level in the study.

**Figure 1:**
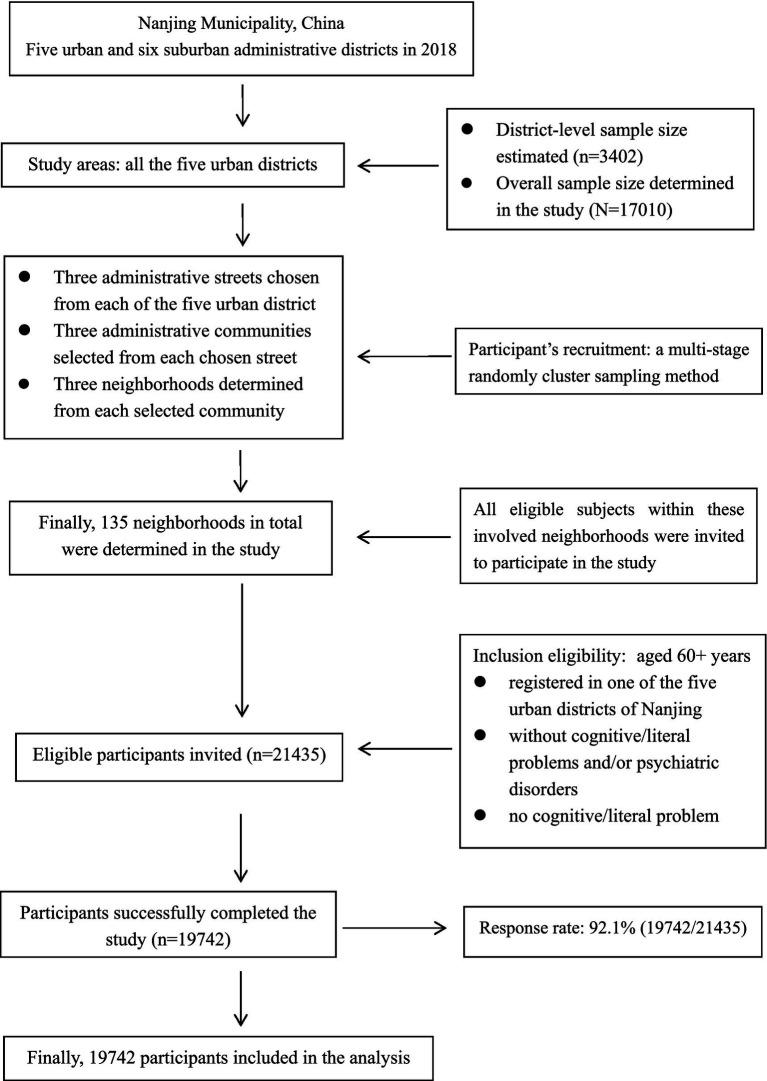
Flow chart of participant’s selection in the study.

In China, there are four levels of official government body, including central government, province/municipality, urban district/rural country, and administrative street/town. However, in the actual situation, a non-government entity, the administrative urban community/rural village is also involved in the administrative system as the lowest and geographically smallest stratum. Typically, a community/village comprises of several neighborhoods. In this study, participants were randomly selected from all the five urban districts using a multi-stage sampling approach. First, three administrative streets were selected from each of the five urban district. Then, three administrative communities were determined within each of the three selected streets. Next, three neighborhoods were chosen from each of the three determined communities. Thus, 135 neighborhoods were finally chosen from all the five urban districts, and all eligible subjects within these selected neighborhoods were invited to participate in the study. Figure 1 displayed the flowchart of participants’ selection.

A written informed consent was provided by each participant. This study was reviewed and approved by The Ethics Committee of Geriatric Hospital of Nanjing Medical University. Data were de-identified before analysis. The methods employed in this study were in line with recommendations by the Declaration of Helsinki.

### Data collection

The information was collected via questionnaire survey, including participant’s age, gender, educational attainment, marital status, income (retirement pension), health insurance, self-reported history of main chronic conditions (including diabetes, coronary heart disease, stroke and dyslipidemia). Meanwhile, BP, body weight and height were objectively measured for each participant.

BP was assessed for each participant with a calibrated sphygmomanometer using Korotkoff sounds based on standardized procedures recommended for Chinese BP measurement (29). BP was measured at least twice for each participant and the mean value was used in this study (29). As for measurement of body weight and height, they were recorded to the nearest 0.1 kilogram and 0.01 meter, respectively, for each participant. Moreover, they were also measured two times, separately, and the means were applied to calculate the body mass index (BMI) with boy weight (kg) divided by height (m^2^) according to the scheme of chronic non-communicable disease and risk factor surveillance in China (30).

### Study variables

#### Outcome variable

The outcome variable was BP status. The diagnosis and classification of BP in this study were based on the Guidelines for the Primary Management of Hypertension in China (29). An individual who had been diagnosed as a HTN patient by a registered physician was classified as “having diagnosed HTN” in the study. On the other hand, a participant was defined as “having un-diagnosed HTN,” if his/her systolic/diastolic BP was assessed ≥140/90 mmHg (29) and he/she was not diagnosed as a HTN patient previously by a doctor. Thus, each participant was categorized as having “normal blood pressure,” “diagnosed HTN” or “un-diagnosed HTN” in the analysis.

#### Independent variable

The independent variable was health insurance status. In our study city, Nanjing, two types of health insurance scheme, UEBMI and URBMI, were established for all urban residents in 2007 and worked well afterwards (25, 31). However, a disparity of benefits exists between URBMI and UEBMI, as UEBMI beneficiaries can get more reimbursement benefits compared with their URBMI counterparts (26, 32). For URBMI, the reimbursement rate was 50% for outpatient visit to community-level primary care service centers, while only 30% for outpatient visit to general hospitals, and moreover there was an RMB360000.0 upper limit of reimbursement per person per year (33). On the other hand, for UEBMI, the reimbursement rate was 70 and 60% for outpatient visit to primary care centers and general hospitals, respectively, and there was no upper limit of reimbursement (34). All the participants were covered by either URBMI or UEBMI in this study, and thus categorized into having “URBMI” or “UEBMI” in the analysis.

#### Covariates

Some covariates were considered in this study. According to findings from the recent China Health and Retirement Longitudinal Study (CHARLS), resident’ socio-demographic characteristics, and status of main chronic conditions were the influencing factors for healthcare seeking behaviors (35). Thus, participants’ socio-demographic characteristics and status of selected chronic conditions were treated as potential confounders in the analysis. Socio-demographic characteristics referred to subject’s age (60–69, 70–79 or 80+ years), gender (men or women), educational attainment (≤6, 7–12 or 13+ schooling years), marital status (“single” or “having a spouse/partner”) and income (“< annually mean value of retirement pension” or “≥ annually mean value of retirement pension”).

The most common abnormal health conditions that might influence residents to seek outpatient service were also adjusted for in the analysis, including excess body weight, diabetes, coronary heart disease, stroke and dyslipidemia. Participants were classified into subgroups of “not excess body weight (BMI < 24 kg/m^2^),” overweight (BMI: 24–27 kg/m^2^)” or “obese (BMI > =28 kg/m^2^)” based on BMI cutoffs recommended specifically for Chinese adults (30). The histories of diabetes, coronary heart disease, stroke and dyslipidemia, one by one, were self-reported by each participant. Thus, a participant was classified as “having the disease (Yes),” if he/she had been clinically diagnosed with one of the corresponding diseases. Otherwise, he/she was classified as “not having the disease (no)” in the analysis.

### Data analysis

Firstly, chi-square test was applied to compare the differences in health insurance status and the prevalence of diagnosed and un-diagnosed HTN by participant’s socio-demographic features. Then, three mixed-effect logistic regression models were introduced to estimate odds ratios (ORs) and 95% confidence intervals (CIs) for examining the association of health insurance with diagnosed and un-diagnosed HTN, separately. Model 1 was a univariate analysis with health insurance as the single explanatory variable and study neighborhood as the random effect. Model 2 was a multivariate analysis with adjustment for participants’ socio-demographic characteristics (age, gender, educational level, marital status, and income) in addition to those included in Model 1. Model 3 was also a multivariate analysis with further consideration of history of chronic conditions (body weight status, self-reported diabetes, self-reported coronary heart disease, self-reported stroke, and self-reported dyslipidemia) in addition to those adjusted for in Model 2. The significance level was set as *p* < 0.05 (two-sided). EpiData 3.1 (The EpiData Association 2008, Odense, Denmark) and SPSS version 20.0 for Windows (SPSS Inc., Chicago, IL, United States) were used to enter and analyze the data.

## Results

### Selected characteristics of participants

Totally, 21,435 older residents within all the 135 participating urban neighborhoods were eligible for this study. Actually, 19,742 successfully completed the study (response rate = 92.1%). There was no difference in age or gender between the respondents and non-respondents. Table 1 showed selected socio-demographic characteristics of participants by health insurance in the study. There were 61.0% subjects aged 60–69 years and 8.3% aged 80+ years, 47.8% of men, 10.9% with 13+ schooling years of educational attainment, 86.2% having a spouse or partner, 69.4% having retirement pension not less than the mean level. The proportions of participants with URBMI and UEBMI were 35.4 and 64.6%, respectively. A significant difference in age, gender, educational level, marital status, or income was observed between the two health insurance schemes, separately.

**Table 1:** Selected socio-demographic and anthropometric characteristics of participants aged 60+ years in urban areas of Nanjing of China by health insurance in this study.

		Participants by health insurance, % (*n*)			
		Total	URBMI^†^	UEBMI^‡^	*p* value*
**Overall**		*N* = 19,742	35.4 (6983)	64.6 (12759)	
**Age (yrs)**					
	60–69	61.0 (12051)	63.1 (4044)	59.9 (7647)	
	70–79	30.7 (6044)	28.9 (2017)	31.6 (4027)	<0.001
	80+	8.3 (1647)	8.0 (562)	8.5 (1085)	
**Gender**					
	Men	47.8 (9442)	40.6 (2837)	51.8 (6605)	<0.001
	Women	52.2 (10300)	59.4 (4146)	48.2 (6154)	
**Educational attainment (schooling years)**					
	0–7	40.8 (8045)	76.0 (5305)	21.5 (2740)	
	7–12	48.4 (9548)	22.2 (1553)	62.7 (7995)	<0.001
	13+	10.9 (2149)	1.8 (125)	15.9 (2024)	
**Marital status**					
	Single	13.8 (2715)	17.9 (1249)	11.5 (1466)	<0.001
	Having a spouse/partner	86.2 (17027)	82.1 (5734)	88.5 (11293)	
**Income**					
	<annually mean value of retirement pension	30.6 (6036)	38.9 (2717)	26.0 (3319)	<0.001
	≥annually mean value of retirement pension	69.4 (13706)	61.1 (4266)	74.0 (9440)	

†URBMI: Urban Resident Basic Medical Insurance for individuals who do not have a regular job, such as students or disabled individuals. ‡ UEBMI: Urban Employment Basic Medical Insurance for individuals who work for the formal or registered sectors. * Chi-square test.

### Prevalence of diagnosed and un-diagnosed HTN

Table 2 displayed the prevalence of diagnosed and un-diagnosed HTN among participants in the study. Among overall participants, the prevalence of diagnosed and un-diagnosed HTN was 47.2% (95%CI = 46.5, 47.9%) and 12.7% (95%CI = 12.2, 13.2%), respectively. Moreover, the prevalence of diagnosed HTN was 38.4% (95%CI = 37.3, 39.6%) and 52.1% (95%CI = 51.2, 53.0%), separately, for residents with URBMI and UEBMI, while the prevalence of un-diagnosed HTN was 18.5% (95%CI = 17.6, 19.4%) and 9.6% (95%CI = 9.1, 10.1%) among subjects with URBMI and UEBMI, respectively. Furthermore, the prevalence of either diagnosed or un-diagnosed HTN differed significantly within health insurance status by each of the selected participant’s socio-demographic characteristics in this study.

**Table 2:** Prevalence of diagnosed and un-diagnosed hypertension among participants aged 60+ years in urban areas of Nanjing of China in this study.

		Prevalence of hypertension, % (*n*/*N*)							
		Diagnosed hypertension				Un-diagnosed hypertension			
		Total	URBMI^†^	UEBMI^‡^	*p* value^*^	Total	URBMI^†^	UEBMI^‡^	*p* value^*^
**Overall**		47.2% (9,328/19742)	38.4 (2,679/6983)	52.1 (6,649/12759)	<0.001	12.7% (2,509/19742)	18.5 (1,289/6983)	9.6 (1,220/12759)	<0.001
**Age (yrs)**									
	60–69	42.8 (5,154/12051)	35.3 (1,555/4404)	47.1 (3,599/7647)		12.4 (1,494/12051)	17.3 (760/4404)	9.6 (734/7647)	
	70–79	54.4 (3,289/6044)	43.8 (884/2017)	59.7 (2,405/4027)	<0.001	13.1 (793/6044)	20.3 (409/2017)	9.5 (384/4027)	<0.001
	80+	53.7 (885/1647)	42.7 (240/562)	59.4 (645/1085)		13.5 (222/1647)	21.4 (120/562)	9.4 (102/1085)	
**Gender**									
	Men	49.3 (4,655/9442)	38.7 (1,099/2837)	53.8 (3,556/6605)	<0.001	12.6 (1,186/9442)	19.2 (544/2837)	9.7 (642/6605)	<0.001
	Women	45.4 (4,673/10300)	38.1 (1,580/4146)	50.3 (3,093/6154)		12.8 (1,323/10300)	18.0 (745/4146)	9.4 (578/6154)	
**Educational attainment (schooling years)**									
	0–7	41.9 (3,371/8045)	37.2 (1972/5305)	51.1 (1,399/2740)		17.1 (1,372/8045)	20.2 (1,072/5305)	10.9 (300/2740)	
	7–12	50.8 (4,855/9548)	42.1 (654/1553)	52.5 (4,201/7995)	<0.001	10.2 (973/9548)	13.5 (210/1553)	9.5 (763/7995)	<0.001
	13+	51.3 (1,102/2149)	42.4 (53/125)	51.8 (1,049/2024)		7.6 (164/2149)	5.6 (7/125)	7.8 (157/2024)	
**Marital status**									
	Single	49.5 (1,343/2715)	40.4 (504/1249)	57.2 (839/1466)	<0.001	14.7 (400/2715)	20.4 (255/1249)	9.9 (145/1466)	<0.001
	Having a spouse/partner	46.9 (7,985/17027)	37.9 (2,175/5734)	51.4 (5,810/11293)		12.4 (2,109/17027)	18.0 (1,034/5734)	9.5 (1,075/11293)	
**Income**									
	<annually mean value of retirement pension	44.1 (2,662/6036)	3,709 (1,029/2717)	49.2 (1,633/3319)	<0.001	13.9 (837/6036)	17.6 (479/2717)	10.8 (358/3319)	<0.001
	≥annually mean value of retirement pension	48.6 (6,666/13706)	38.7 (1,650/4266)	53.1 (5,016/9440)		12.2 (1,672/13706)	19.0 (810/4266)	9.1 (862/9440)	

† URBMI: Urban Resident Basic Medical Insurance for individuals who do not have a regular job, such as students or disabled individuals. ‡ UEBMI: Urban Employment Basic Medical Insurance for individuals who work for the formal or registered sectors. * Chi-square test.

### The associations of health insurance with diagnosed and un-diagnosed HTN

Table 3 presented the associations of health insurance with diagnosed and un-diagnosed HTN among participants. Among overall participants, those who had UEBMI were more likely to have HTN identified (OR = 1.53; 95%CI = 1.44, 1.63) and at lower odds to experience un-diagnosed HTN (OR = 0.58; 95%CI = 0.53, 0.64) compared to their counterparts with URBMI. Moreover, with control of participant’s socio-demographic characteristics and selected chronic conditions, the associations of health insurance with diagnosed and un-diagnosed HTN attenuated but still remained significant.

**Table 3:** The association of health insurance with diagnosed and un-identified hypertension among urban residents aged 60+ years in regional China.

		Category of health insurance	Odd ratios (95% confidence interval)							
Variables			Diagnosed hypertension				Un-diagnosed hypertension			
			% (*n*/*N*)	Model 1 *	Model 2 #	Model 3 $	% (*n*/*N*)	Model 1 *	Model 2 #	Model 3 $
Overall		URBMI^†^	38.4 (2,679/6983)	1	1	1	18.5 (1,289/6983)	1	1	1
		UEBMI^‡^	52.1 (6,649/12759)	1.53 (1.44, 1.63)	1.50 (1.40, 1.61)	1.20 (1.11, 1.29)	9.6 (1,220/12759)	0.58 (0.53, 0.64)	0.70 (0.63, 0.78)	0.68 (0.61, 0.76)
Age (yrs)										
	60–69	URBMI^†^	35.3 (1,555/4404)	1	1	1	17.3 (760/4404)	1	1	1
		UEBMI^‡^	47.1 (3,599/7647)	1.46 (1.35, 1.58)	1.47 (1.33, 1.61)	1.20 (1.09, 1.33)	9.6 (734/7647)	0.61 (0.54, 0.68)	0.77 (0.67, 0.89)	0.76 (0.66, 0.87)
	70–79	URBMI^†^	43.8 (884/2017)	1	1	1	20.3 (409/2017)	1	1	1
		UEBMI^‡^	59.7 (2,405/4027)	1.59 (1.41, 1.79)	1.55 (1.35, 1.78)	1.20 (1.04, 1.38)	9.5 (384/4027)	0.55 (0.47, 0.65)	0.62 (0.52, 0.76)	0.61 (0.50, 0.74)
	80+	URBMI^†^	42.7 (240/562)	1	1	1	21.4 (120/562)	1	1	1
		UEBMI^‡^	59.4 (645/1085)	1.61 (1.28, 2.02)	1.57 (1.21, 2.03)	1.20 (0.92, 1.57)	9.4 (102/1085)	0.51 (0.37, 0.70)	0.61 (0.42, 0.87)	0.58 (0.40, 0.84)
Gender										
	Men	URBMI^†^	38.7 (1,099/2837)	1	1	1	19.2 (544/2837)	1	1	1
		UEBMI^‡^	53.8 (3,556/6605)	1.61 (1.46, 1.77)	1.48 (1.33, 1.66)	1.14 (1.02, 1.28)	9.7 (642/6605)	0.59 (0.51, 0.67)	0.64 (0.55, 0.75)	0.61 (0.52, 0.71)
	Women	URBMI^†^	38.1 (1,580/4146)	1	1	1	18.0 (745/4146)	1	1	1
		UEBMI^‡^	50.3 (3,093/6154)	1.44 (1.32, 1.56)	1.53 (1.38, 1.69)	1.26 (1.13, 1.40)	9.4 (578/6154)	0.57 (0.50, 0.64)	0.77 (0.67, 0.90)	0.77 (0.66, 0.90)

*Model 1: uni-variate mixed-effect logistic regression analysis with health insurance as the independent variable and neighborhood as the random effect. # Model 2: multi-variate logistic regression analysis, with adjustment for participants’ age, gender, educational level, marital status, and income as well as neighborhood-level clustering effects. $ Model 3: multi-variate logistic regression analysis, with consideration of body weight status, self-reported diabetes, self-reported coronary heart disease, self-reported stroke, self-reported dyslipidemia in addition to those adjusted for in Model 2. † URBMI: Urban Resident Basic Medical Insurance for individuals who do not have a regular job, such as students or disabled individuals. ‡ UEBMI: Urban Employment Basic Medical Insurance for individuals who work for the formal or registered sectors.

Furthermore, interesting scenarios were observed when stratification analysis was conducted by age and gender. The relationship between health insurance and diagnosed HTN was significant among each stratum of age and gender with consideration of participants’ socio-demographic characteristics. However, such an association became non-significant for participants aged 80+ years (UEBMI vs. URBMI: OR = 1.20; 95%CI = 0.92, 1.57) with additional control of chronic conditions in the analysis. On the other hand, the scenario of relationship between health insurance and un-diagnosed HTN for participants within each age and gender sub-group was the same as that among overall subjects regardless of adjustment for socio-demographic characteristics or chronic conditions additionally.

## Discussion

This community-based study was developed mainly to explore the potential role of health insurance for early identifying HTN among older residents through investigating the associations of health insurance with diagnosed and un-diagnosed HTN in regional China. It was examined that, compared to their URBMI counterparts, UEBMI beneficiaries were more likely to have HTN diagnosed if they had the disease, which suggested that favorable health insurance scheme might play a positive role in early-identification of HTN for older individuals in China.

Our study reported that beneficiary-friendly health insurance scheme (UEBMI vs. URBMI) was in a positive relation to diagnosed HTN but a negative link with un-diagnosed HTN among urban residents aged 60+ years in China. Findings from our study were in line with those reported in previous studies from USA and China, although participants were adults aged 20–64 or 45+ years in the previous studies but subjects aged 60+ years in ours (20–23). Moreover, our study also found that such associations of health insurance with diagnosed and un-diagnosed HTN remained for participants stratified by gender and age. Therefore, our findings added new evidence to the existing literature that adequate health insurance scheme may be favorable for early identification of HTN among not only non-older adults but also the retirees aged 60+ years. This implies that the favorable association between health insurance and identification of HTN may hold steadily for adult residents with different age.

The potential mechanisms behind the observed associations of health insurance with diagnosed and un-diagnosed HTN in this study might be addressed through participants’ health service seeking behavior and the probability of blood pressure measured by doctors. Under a situation that each person visits a hospital or primary care center will have his/her blood pressure measured by a doctor, it is reasonable that an individual who is at a higher odds to visit hospitals or primary care centers will tend to have blood pressure measured. In China, residents aged 35+ years would have their blood pressure assessed by doctors at their visits to hospitals or primary care centers since 2012 (10). Meanwhile, it has been documented consistently that the favorable health insurance scheme may increase the likelihood for people to seek health service at hospitals or primary care centers in China (11–19, 36). Thus, beneficiaries with the favorable health insurance scheme (e.g., UEBMI in our study) were more likely to visit hospitals or primary care centers for seeking health service, and consequently had their blood pressure measured by doctors, and then had hypertension diagnosed by a doctor if they had the condition. This may be the fundamental explanation for the positive association of health insurance with diagnosed HTN, and the negative relationship between health insurance and un-diagnosed HTN among residents aged 60+ years in our study.

Considering that a high prevalence of HTN among older people aged 60+ years in China, a fast-aging society in the world (3, 5), there will be more and more HTN patients in the future. Thus, it is a big challenge for China to identify hypertensive individuals as early as possible, as no diagnosis means no treatment for HTN patients. Fortunately, findings in our study suggested that favorable health insurance may be an effective pathway for identifying hypertensive people early at a population level. When a universal coverage of health insurance almost has been achieved today in China (27), the next step shall be to improve reimbursement rate of UEBMI and, especially, URBMI for the purpose to have hypertensive people identified as early as possible. From a population health perspective, this is the specific important implication of our study for policy makers involved in HTN prevention and control in China.

Several strengths need to be noted in our study. Firstly, participants were randomly selected from 135 neighborhoods in all urban districts of a typical mega-city in eastern China, and the response rate was 92.1%. These imply the representativeness of participants and generalizability of the findings. Secondly, subjects were limited to older residents aged 60+ years, as they had a very heavy disease burden caused by HTN and no study was available on health insurance and HTN identification among them. It is of particular meaningfulness to identify HTN individuals early and subsequently to provide them with adequate treatment for the purpose to reduce HTN burden for older people. Thirdly, findings were consistent between overall and each stratum of participants by either age or gender, suggesting a solid relationship between health insurance and identification of HTN among older residents in China. Finally, this study has important public health implications for population-level HTN prevention and control.

Limitations of the study shall also be mentioned. The first, although BP was measured according to the Guidelines for the Primary Management of Hypertension in China (29), it was a filed-measurement in an epidemiological survey, not a clinical assessment by a physician at hospitals/primary care centers. Thus, hypertensive individuals defined in this study were actually those determined by epidemiologists not physicians, and some of them might not completely meet the concept of clinical HTN patients (37). This may result in over-estimating the un-diagnosed HTN prevalence in this study. The second, information on income (retirement pension), and chronic disease history were self-reported by participants. Therefore, potential recall bias might exist. The third, although the most common chronic conditions were adjusted for as covariates in the analysis, some potential abnormal conditions, e.g., COPD, cancers, etc., were not considered in the study. Fourth, due to the nature of a cross-sectional study, no causal association of health insurance with diagnosed ad un-diagnosed HTN could be inferred from this study. So findings observed in our study shall be interpreted prudently. In future, well-designed longitudinal population-based studies are encouraged to further investigate the potential causal impact of health insurance on HTN identification within not only retirees but also residents within other age-groups in China and other countries.

## Conclusion

Favorable health insurance may be a pathway for identifying hypertension among residents aged 60+ years in urban areas of China. This study is of important significance that, from a population health perspective, hypertension may be identified early through favorable health insurance policies for older people in China.

## Data availability statement

The original contributions presented in the study are included in the article/supplementary material, further inquiries can be directed to the corresponding authors.

## Ethics statement

The studies involving humans were approved by the Ethics Committee of Geriatric Hospital of Nanjing Medical University. The studies were conducted in accordance with the local legislation and institutional requirements. Written informed consents were obtained from all participants.

## Author contributions

YD: Conceptualization, Investigation, Writing – original draft, Writing – review & editing. HG: Conceptualization, Investigation, Writing – original draft, Writing – review & editing. SL: Writing – original draft, Writing – review & editing. HX: Writing – original draft, Writing – review & editing. FL: Writing – original draft, Writing – review & editing. WT: Writing – original draft, Writing – review & editing. SJ: Writing – original draft, Writing – review & editing. ST: Writing – original draft, Writing – review & editing. TD: Writing – original draft, Writing – review & editing. YX: Conceptualization, Funding acquisition, Investigation, Methodology, Project administration, Resources, Supervision, Writing – original draft, Writing – review & editing. FX: Conceptualization, Formal analysis, Funding acquisition, Investigation, Methodology, Project administration, Resources, Supervision, Writing – original draft, Writing – review & editing.

## References

[1] World Health Organization. Health Topics. Hypertension. Available at: https://www.who.int/health-topics/hypertension#tab=tab_1 (Accessed May 03, 2024).

[2] Poulter NR Prabhakaran D Caulfield M. Hypertension. Lancet. (2015) 386:801–12. doi: 10.1016/S0140-6736(14)61468-9 25832858

[3] Zhang M Wu J Zhang X Hu CH Zhao ZP Li C. Prevalence and control of hypertension in adults in China, 2018. Zhonghua Liu Xing Bing Xue Za Zhi. (2021) 42:1780–9. doi: 10.3760/cma.j.cn112338-20210508-00379 34814612

[4] Li S Chen Z Wang Z Wang X Zhang L Dong Y. The hypertension status of the elder population in China. Chinese J Hypertens. (2019) 27:140–8. doi: 10.16439/j.cnki.1673-7245.2019.02.010

[5] Lu J Lu Y Wang X Li X Linderman GC Wu C. Prevalence, awareness, treatment, and control of hypertension in China: data from 1.7 million adults in a population-based screening study (China PEACE million persons project). Lancet. (2017) 390:2549–58. doi: 10.1016/S0140-6736(17)32478-9 29102084

[6] National Statistical Bureau. The 5th bulletin of the 7th census of China. Available at: http://www.stats.gov.cn/ztjc/zdtjgz/zgrkpc/dqcrkpc/ggl/202105/t20210519_1817698.html (Accessed February 17, 2023).

[7] Tao S Wu X Liu G Liu L. Approaches for population-based hypertension identification and treatment in China. Chinese J Hypertens. (1994) 2:1–3. doi: 10.16439/j.cnki.1673-7245.1994.01.001

[8] Zheng Y Li D Li X. Implementation effect of blood pressure measurement for people aged 35+ years at their visit to hospitals. Shanghai J Prev Med. (2001) 2:98–9. doi: 10.19428/j.cnki.sjpm.2001.02.031

[9] Wang P Wan Y. Feasibility of policy of blood pressure measurement for patients aged 35+ years at their visit to hospital. Chinese J Health Educ. (2003) 19:17–20. doi: 10.16168/j.cnki.issn.1002-9982.2003.01.006

[10] The State Council of China. The twelfth five-year plan on development of health service. Available at: http://www.gov.cn/zwgk/2012-10/19/content_2246908.htm (Accessed October 25, 2022).

[11] Yu D Li X Feng S. Has health insurance changed people’s healthcare seeking behaviors? Evidence from the urban residents’ health insurance policy in China. J Guangxi Cadres Coll Econ Manag. (2019) 31:19–24. doi: 10.3969/j.issn.1008-8806.2019.01.004

[12] Cao Q Song H. Analysis on the policy effect of medical insurance on out-patients among urban residents: evidence from the China health and retirement longitudinal study (CHARLS). Soc Secur Stud. (2022) 4:23–31. doi: 10.3969/j.issn.1674-4802.2022.04.003

[13] Chen L Wang Y. Influencing factors of healthcare seeking behaviors among elderlies. J Trad Chin Med Manag. (2015) 23:18–21. doi: 10.16690/j.cnki.1007-9203.2015.18.088

[14] Wang S. Analyzing health seeking behavior of Chinese residents and their influencing factors: based on CHNS data. Northwest Popul. (2015) 3:32–6. doi: 10.3969/j.issn.1007-0672.2015.03.007

[15] Zheng L. Has health insurance system changed healthcare seeking behavior?-evidence from Chinese CHNS. Fisc Stud. (2017). 2:84–97. doi: 10.19477/j.cnki.11-1077/f.2017.02.008

[16] Zeng B Yuan Z Fang Y. Healthcare seeking behavior among Chinese older adults: patterns and predicting factors. Chin J Health Stat. (2020) 37:199–205. 10.3390/ijerph18062969 PMC7998758 33799366

[17] Huang C Miao C Shi S Yin Y Huang X Li B. Analysis of the empty nesters’ healthcare seeking behavior and influencing factors in China. Modern Prev Med. (2022) 49:295–8.

[18] Wang J Pei Y Zhong R Wu B. Outpatient visits among older adults living alone in China: does health insurance and City of residence matter? Intl J Environ Res Public Health. (2020) 17:4256. doi: 10.3390/ijerph17124256, PMID: 32549227 PMC7344973

[19] Fan G Deng Z Wu X Wang Y. Medical insurance and health equity in health service utilization among the middle-aged and older adults in China: a quantile regression approach. BMC Health Serv Res. (2020) 20:553. doi: 10.1186/s12913-020-05423-y, PMID: 32552901 PMC7302153

[20] Schober SE Makuc DM Zhang C Kennedy-Stephenson J Burt V. Health insurance affects diagnosis and control of hypercholesterolemia and hypertension among adults aged 20-64: United States, 2005-2008. NCHS Data Brief. (2011) 57:1–8. 21592420

[21] Feng XL Pang M Beard J. Health system strengthening and hypertension awareness, treatment and control: data from the China health and retirement longitudinal study. Bull World Health Organ. (2014) 92:29–41. doi: 10.2471/BLT.13.124495, PMID: 24391298 PMC3865551

[22] Fowler-Brown A Corbie-Smith G Garrett J Lurie N. Risk of cardiovascular events and death--does insurance matter? J Gen Intern Med. (2007) 22:502–7. doi: 10.1007/s11606-007-0127-2, PMID: 17372800 PMC1829431

[23] Hogan DR Danaei G Ezzati M Clarke PM Jha AK Salomon JA. Estimating the potential impact of insurance expansion on undiagnosed and uncontrolled chronic conditions. Health Affairs (Millwood). (2015) 34:1554–62. doi: 10.1377/hlthaff.2014.1435, PMID: 26355058 PMC12266343

[24] State Council of China (1998). Decisions of the state council on establishing the urban employee essential medical scheme. Available at: http://www.ldzc.com/html/law/fagui/20.html (Accessed February 15, 2023).

[25] State Council of China (2007). Instructions on establishing the urban employee essential medical scheme. Available at: http://www.gov.cn/zwgk/2007-07/24/content_695118.htm (Accessed February 15, 2023).

[26] Huang J Yuan L Liang H. Which matters for medical utilization equity under universal coverage: insurance system, region or SES. Intl J Environ Res Public Health. (2020) 17:4131. doi: 10.3390/ijerph17114131, PMID: 32531889 PMC7312584

[27] National Health Commission of China. China health statistical yearbook. Beijing: Beijing Union Medical University Press (2018).

[28] Nanjing Municipal Bureau of Statistics. Available at: http://tjj.nanjing.gov.cn/material/njnj_2019/renkou/3-1.htm (Accessed February 17, 2023).

[29] Writing Group. The guidelines for the primary Management of Hypertension in China (2014). Chin. J Health Manag. (2015) 9:10–30. doi: 10.3760/cma.j.issn.1674-0815.2015.01.004

[30] Zhang M Li Y Huang Z Deng Q Zhao Z Li C. Scheme of the Chinese chronic non-communicable disease and risk factor surveillance. Chin. J Prev Med. (2018) 52:191–4. doi: 10.3760/cma.j.issn.0253-9624.2018.02.015 29429277

[31] Jiangsu Provincial Government. Guidance for the establishment of basic medical insurance system for urban residents. Available at: http://www.jiangsu.gov.cn/art/2007/4/5/art_46143_2543693.html (Accessed February 15, 2023).

[32] Yip WC Hsiao WC Chen W Hu S Ma J Maynard A. Early appraisal of China's huge and complex health-care reforms. Lancet. (2012) 379:833–42. doi: 10.1016/S0140-6736(11)61880-1, PMID: 22386036

[33] Nanjing Municipal Bureau of Healthcare Security. Municipal guidance for integration of URBMI for urban and rural residents. Available at: http://ybj.nanjing.gov.cn/gkml/201903/t20190325_1475797.html (Accessed February 15, 2023).

[34] Nanjing Municipal Bureau of Healthcare Security. Municipal regulation on urban employee health insurance. Available at: http://ybj.nanjing.gov.cn/ztzl/czzgyb/201903/t20190329_1492755.html (Accessed February 15, 2023).

[35] Zeng Y Wan Y Yuan Z Fang Y. Healthcare-seeking behavior among Chinese older adults: patterns and predictive factors. Int J Environ Res Public Health. (2021) 18:2969. doi: 10.3390/ijerph18062969, PMID: 33799366 PMC7998758

[36] Wagstaff A Lindelow M Gao J Xu L Qian J. Extending health insurance to the rural population: an impact evaluation of China’s new cooperative medical scheme. J Health Econ. (2009) 28:1–19. doi: 10.1016/j.jhealeco.2008.10.007, PMID: 19058865

[37] Yang X Zhang W Shuai W Chen H Zhou W Bao H. The clinical diagnose rate of hypertension of the subjects with newly-diagnosed hypertension identified by epidemiological study. Chin J Hypertens. (2018) 26:1026–9. doi: 10.16439/j.cnki.1673-7245.2018.11.011

